# The global prevalence of oral leukoplakia: a systematic review and meta-analysis from 1996 to 2022

**DOI:** 10.1186/s12903-023-03342-y

**Published:** 2023-09-06

**Authors:** Chang Zhang, Bingjie Li, Xiamei Zeng, XiaoSheng Hu, Hong Hua

**Affiliations:** 1 Department of Oral Medicine, Peking University School and Hospital of Stomatology & National Center of Stomatology & National Clinical Research Center for Oral Diseases & National Engineering Laboratory for Digital and Material Technology of Stomatology & Beijing Key Laboratory of Digital Stomatology & Research Center of Engineering and Technology for Computerized Dentistry Ministry of Health & NMPA Key Laboratory for DentalMaterials, Haidian District, No.22, Zhongguancun South Avenue, Beijing, 100081 People’s Republic of China; 2 Xiamen Key Laboratory of Stomatological Disease Diagnosis and Treatment, Stomatological Hospital of Xiamen Medical College, Xiamen, China

**Keywords:** Oral Leukoplakia, Global prevalence, Systematic review, Meta-analysis

## Abstract

**Background:**

Oral leukoplakia(OLK) is a common oral potentially malignant disorder. The global prevalence of solely OLK was published in 2003, while the prevalence varied among different studies. In recent years, large-scale summary and definition-related analyses obtain insufficient attention. This study aimed to perform a systematic review of prevalence studies of oral leukoplakia and assess predisposing factors of its occurrence.

**Methods:**

The search terms ("Oral leukoplakia" OR OLK OR leukoplakia) AND (prevalence OR incidence OR epidemiology) were searched in databases (Pubmed, Embase, Scopus, and Web of Science) for OLK studies published from January 1996 until December 2022. The estimated prevalence calculation and risk of bias analysis used STATA 16.0.

**Results:**

We obtained 69 studies, including 1,263,028 participants, from 28 countries, and 6 continents. The prevalence was 1.39%, varying from 0.12 to 33.33%. The overall pooled estimated prevalence of OLK was 2.23% for population-based studies, 1.36% for clinic-based population studies, and 9.10% for specific populations. The pooled prevalence in different continents ranged from 0.33 to 11.74% with a statistical difference in the population-based calculation. The estimated prevalence of OLK was higher in males than in females. Those who smoked and consumed alcohol had a higher prevalence than those who did not.

**Conclusion:**

Combining data from 69 published studies, the prevalence of OLK was determined as 1.39% and the pooling estimated global prevalence was 3.41%. The prevalence was relatively consistent and stable across different continents and different definitions. A higher pooled estimated prevalence was found among males, those aged over 60 years old, smokers, and alcohol consumers. The results from the included studies in this systematic review revealed that the prevalence was relatively consistent and stable across various definitions and continents, which may help in developing global treatment and prevention strategies for oral leukoplakia.

**Supplementary Information:**

The online version contains supplementary material available at 10.1186/s12903-023-03342-y.

## Introduction

Oral leukoplakia (OLK) as defined by the World Health Organization (WHO) Collaborating Centre in 2020 as “White plaques of questionable risk having excluded (other) known diseases that carry no increased risk for cancer” [1]. Oral leukoplakia is generally an asymptomatic disorder of the mucosa and is a common oral potentially malignant disorder (OPMD) [1, 2]. The pooled proportion of malignant transformation (MT) was 3.5–9.8%, with the rate varying between 0.13% and 40.8% [3–5]. The annual MT rate is reported as 1.56% [6]. Once oral squamous cell carcinoma (OSCC) occurs, the patients’ 5-year survival rate drops sharply to 50–66% [7]. This not only endangers the patient's life and physical and mental health, but also affects their appearance, causing disability, maxillofacial deformity, and a serious social burden. The reported rates of malignant transformation to OSCC from oral potentially malignant disorders (OPMD) range from 3 to 50%, in which OLK occupies 17–35% [8, 9]. Timely detection, early diagnosis, close monitoring, and treatment management of patients with OLK are imperative.

The definition of leukoplakia was proposed in 1978 by WHO as "A white patch or plaque that cannot be characterized clinically or pathologically as any other disease" [10]. In 1984, the Malmo Conference added " not associated with any physical or chemical causative agent except use of tobacco" [11]. By 1996, the definition was used widely [12], and was formally published in 1997 [13]. Since 2005, the commonly used definition in published studies has been “A predominantly white plaque of questionable risk having excluded (other) known diseases or disorders that carry no increased risk for cancer”, which was published officially in 2007 by the WHO Collaborating Centre. The “risk of cancer development” was emphasized, and was defined as OPMD [14]. The Working Group reiterated the phrase "risk of cancer development" after 2015, and the 2020 workshop on OPMD adhered to this justification. [1]. The diagnosis of OLK depends on irreversible and non-scrapable lesions with the clinical and histological exclusion of other diseases [15]. A biopsy is necessary for definitive diagnosis and risk analysis, and it could detect simple hyperplasia or epithelial dysplasia [9]. Generally, the higher the grade of dysplasia, the higher the risk of cancer [16].

Given that the definition of OLK have evolved multiple times and that the diagnosis is exclusionary, the accurate diagnosis of OLK and predicting malignant transformation remains a challenge in the clinic. Although a number of previous epidemiological studies on OLK have been published, the accurate prevalence is still controversial and lacks geographical and population stratification analysis. The study methods used mainly included house to house surveys, clinical studies, and general investigation in a specific organization or place (such as a company or a school). The description and statistics of the target population, sample size calculation, sampling method, recruitment, diagnosis measure, statistical analysis, confounding factors/subgroups/differences were insufficient. The prevalence varied widely and most came from single center analyses. Currently, the global prevalence of OLK lacks support from epidemiological data. A small number of published reports showed highly heterogeneous results. The overall prevalence of OPMD worldwide was 4.47%, with OLK ranking second with 4.11% [17]. In a study published in 2003, the pooled estimated prevalence of OLK was 1.5% (inverse variance) and 2.6% (random) with no gender predilection [18]. A systematic review claimed that patients less than 40 years old represented 5–76.7% of cases [19]. In recent years, no large-scale summary and definition-related analysis has been published. The majority of meta-analyses and systematic reviews have focused on the rate and risk factors related to malignant transformation. There is also no literature that further explores the prevalence of OLK in different regions and populations. Therefore, the present article aims to review the prevalence of OLK reported from 1996 to 2022, and to classify the research into different groups by continent, definition, age, and living habits.

## Materials and methods

### Protocol registration

This systematic review and meta-analysis complied with the Preferred Reporting Items for Systematic Reviews and Meta-analysis Protocols (PRISMA-P) reporting guidelines [18]. It was registered on the PROSPERO website (www.crd.york.ac.uk/PROSPERO). CRD42021279108 code was assigned.

### Search strategy

According to the PRISMA-P system standard, a literature search was carried out [20]. Two authors (ZC and LBJ) searched for studies published from January 1996 to December 2022 in four databases: PubMed, Embase, Scopus, and Web of science. The search was conducted in February 12th, 2022 and updated in April 10, 2023.

The search keywords/strategy were ("oral leukoplakia" OR OLK OR leukoplakia) AND (prevalence OR incidence OR epidemiology). The search content includes titles, keywords, and abstracts in Embase, Scopus, topic in Web of science, and all field in Pubmed. Two authors independently completed the retrieval of the studies from the four databases. After the preliminary screening, the full text of each article was consulted, downloaded, and sorted together by Endnote. Strict screening was carried out according to the inclusion and exclusion criteria, the study population, and information containing the survey method, the definition of OLK, examination, biopsy, sex, age, smoking, alcohol consumption, betel nut use, country, continent, and district (urban/rural) were evaluated.

### Eligibility criteria

The inclusion criteria for the literature searched were as follows: (1) Original survey with cross-section, case–control, or cohort design by title and abstract screening; (2) Observational study by title and abstract screening; (3) The literature must report the numbers of patients with OLK and total population, to provide sufficient data to calculate the prevalence and possible risk factors inducing OLK. We recorded information about geography, sex, age, living habits (including smoking and alcohol consumption), the definition of OLK, tools of examination, examination standard, and biopsy proposition. If several published studies used the same population database, the study with the largest data was selected.

The exclusion criteria were as follows: (1) Review articles, case reports, agreements, communications, personal opinions, letters, posters, conference abstracts, or laboratory studies were excluded; (2) The data is insufficient, vague, or contains errors that cannot support the statistical analysis; (3) The subjects of the study are non-OLK patients, such as a population with HIV/EBV/HCV infection, vocal cord leukoplakia /cervical leukoplakia /nasal leukoplakia, congenital disease such as dyskeratosis congenita, or research about carcinoma, dysplasia, or only the proportion of OLK in OPMD; (4)The population came from oral medicine or surgery or radiology only in dentistry, and (5) Non-English language literature.

### Study selection

Three authors (ZC, LBJ, and ZXM) individually assessed the eligibility of all the retrieved studies. The studies titles and abstracts of all the studies derived from the search were screened together. When the information in the title or the abstract was insufficient for exclusion, the studies’ full text was reviewed for the final decision on selection of the study. Disagreements regarding the included studies were resolved by collective discussion among the three authors. If the three authors still could not reach an agreement, they consulted another author (HXS or HH).

### Data items and the data collection process

The recorded data of the studies that met the incluson criteria included author, published year, country, continent, sex, age, diagnosis, sample size, estimated prevalence, and risk factors. The prevalence of OLK was calculated by the number of OLK cases as the numerator, and the survey population as the denominator. Data were analyzed using STATA 16.0(Stata Corporation, Texas, USA) for the meta-analysis. We used the estimated prevalence(ES) and 95% confidence interval (CI) to determine statistics of prevalence, and used I^2^ to study the statistical heterogeneity with the random-effect model.

### Evaluation of quality and risk of bias

The risk of bias of the included studies was independently assessed using the Joanna Briggs Institute critical appraisal instrument for studies reporting prevalence data by two authors (ZC and LBJ) [21]. Any ambiguity was discussed and resolved. According to the above appraisal instrument, we analyzed nine items in total (Supplement 2): (1) Was the sample frame appropriate to address the target population? (2) Were the study participants sampled in an appropriate way? (3) Was the sample size adequate? (4) Were the study subjects and the setting described in detail? (5) Was the data analysis conducted with sufficient coverage of the identified sample? (6) Were the diagnostic criteria clearly elaborated? (7) Was the condition measured in a standard, reliable way for all participants? (8) Was there appropriate statistical analysis? (9) Was the response rate adequate, and if not (< 70%), was the low response rate managed appropriately? Each of the nine items in the literature quality evaluation received a score. If the item was low risk, it received a value of 0, otherwise, it received a score of 1. The overall risk of bias was reflected by the total score. We categorized the risk of bias as low, medium, or high (ST1).

### Statistical analysis

The prevalence were analysed by STATA 16.0(Stata Corporation, Texas, USA) using the mataprop command and pooling prevalence with Freeman-Tukey double arcsine transformation. The ES was calculated using a random effect model. The level of significance was set at *P*-value < 0.05 and heterogeneity between the studies was evaluated using I^2^ tests. The preliminary meta-regression analysis aimed to investigate how the population, definition, risk bias, and publication year affected heterogeneity. The ES in different population and definition was calculated. Subgroup analyses were conducted according to underlying factors including age, sex, continent, district, smoking and drinking habits that could impact prevalence. Additional subgroup analysis was performed to determine the impact of bias risk on prevalence. Heterogeneity intra-group and between groups was analyzed. I^2^ < 50% was considered as low heterogeneity. The sensitivity analysis was conducted by leaving out one study at a time to assess the stability of the overall prevalence.

## Results

### Literature search and study selection

**Fig. 1:**
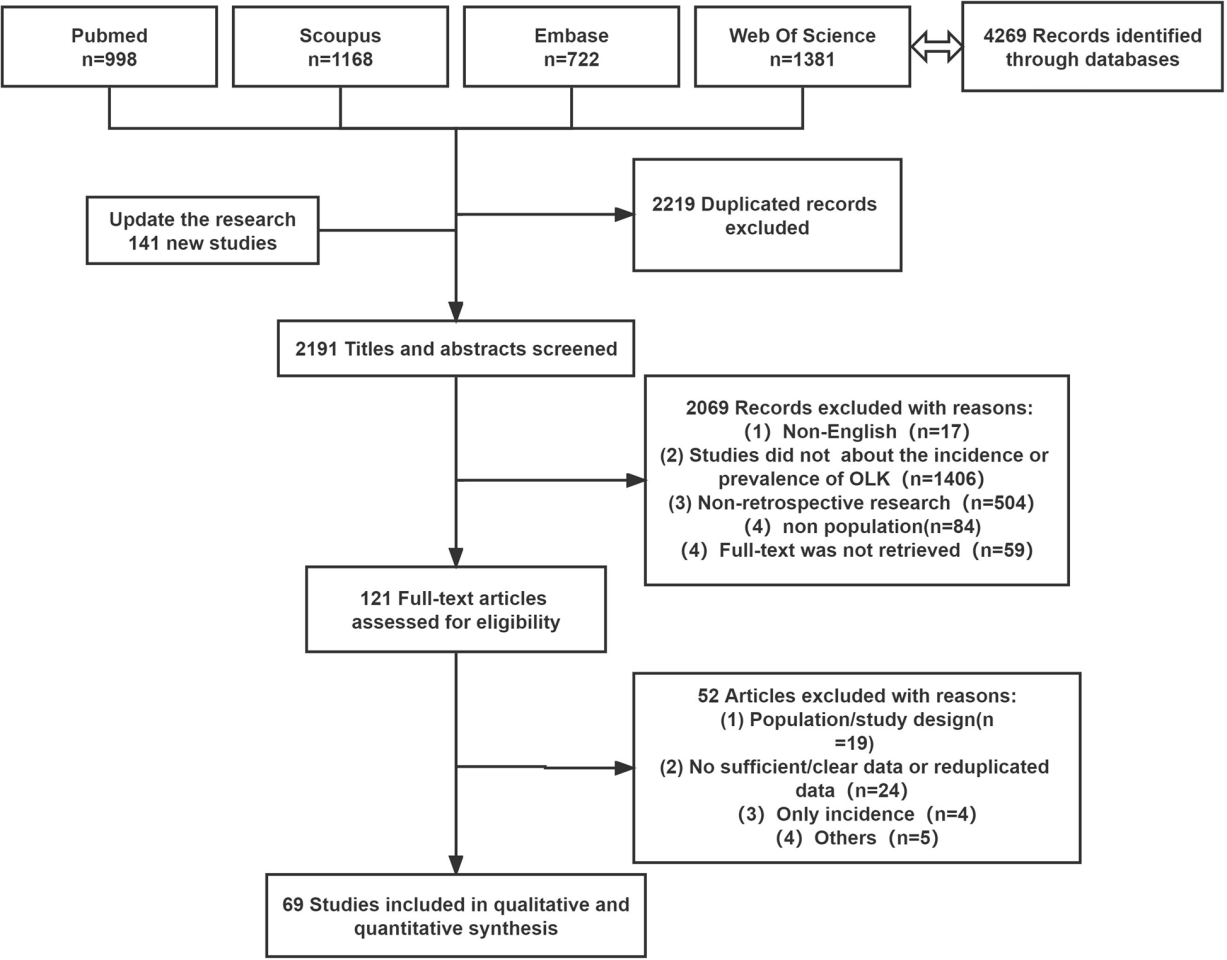
The search flow chart

The flow chart describing the results of the literature search and screening is shown in Fig. 1. We searched 4,269 articles at first. The duplicate studies were excluded by Endnote and artificial examination, then a final 2050 study titles and abstracts were screened. The search was updated in 2023 and 141 new studies added the analysis. Excluding non-professional, non-English literature, non-retrospective literature, and non-research literature on the prevalence of leukoplakia, a total of 159 articles might be eligible, with 121 full-texts accessible. Strict screening and evaluation were conducted according to the inclusion criteria, and 69 studies were finally included in the study. (Fig. 1).

### Overall prevalence

**Fig. 2:**
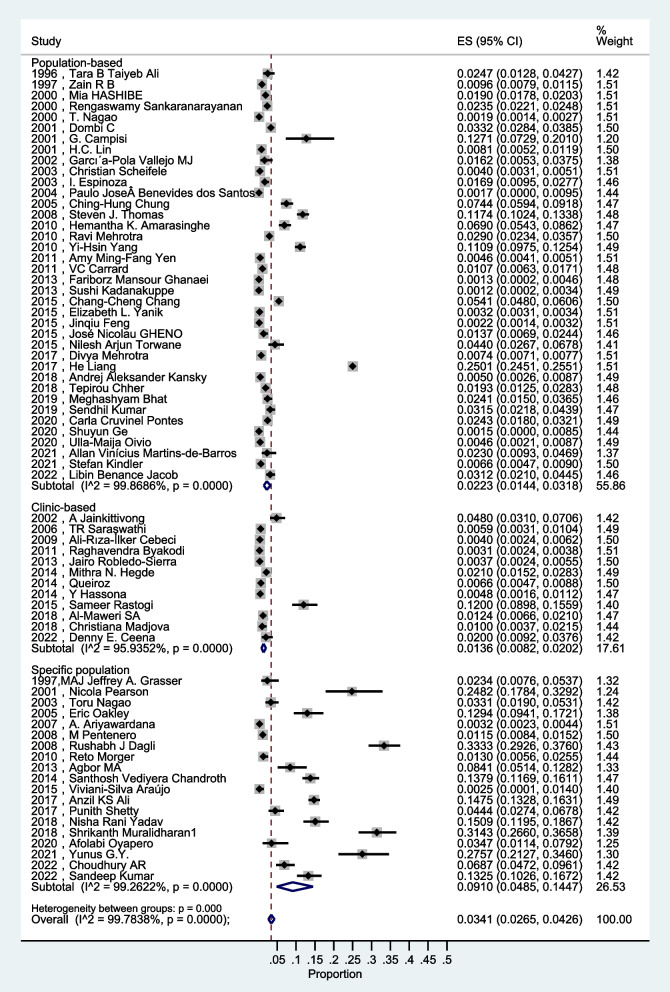
The overall pooled estimated prevalence of OLK in 3 population groups

These 69 studies published from 1996 to 2022 covered 1,263,028 participants, including 17,524 patients with OLK, giving a total prevalence of 1.39%. The random-effects overall estimated prevalence of OLK was 3.41% (95% CI, 2.65–4.26%) with high heterogeneity (I^2^ = 99.78%; *P* < 0.001) (Fig. 2). These 69 studies encompassed 28 countries and spanned the six continents of Asia, Africa, Europe, Oceania, South and North America. According to the source of the populations, the studies were divided into three groups, namely, population-based studies (house to house survey), clinic-based population studies (hospital survey), and specific population studies targeting populations such as soldiers, workers, fishermen, and students. The heterogeneity among 3 population sources of the studies showed significant statistical differences (*P* < 0.001), while excluding the specific population studies the heterogeneity between population-based studies and clinic-based studies showed *P* = 0.098.

### Population-based studies

Table 1 lists the basic information provided in the included population-based studies. In these 38 studies, there were 16,020 patients with OLK out of 1,187,189. The sample size ranged from 118 to 470,266 in 38 studies. The prevalence was 1.35%, ranging from 0.12% to 25.01%. These 38 studies covered the 6 continents of Asia, Africa, Europe, Oceania, South and North America. Twenty-three studies were from Asia, six were from Europe, two were from North America, five were from the South America, one was from Oceania, and one was from Africa. Among the Asian studies, 39% came from India, and separate analysis showed no difference of prevalence between the Indian and other Asian studies.

**Table 1:** The basic information of population-based study

Publish year Authors	Continent	Country	Total	OLK	Definition	Examination	Biopsy	Age
Tara B Taiyeb Ali 1996 [22]	Asia	Malaysia	486	12	WHO 1980	yes	no	≥ 60
Zain R B 1997 [23]	Asia	Malaysia	11,697	112	WHO 1980	yes	no	20–115
Mia HASHIBE 2000 [24]	Asia	India	48,700	927	Other	yes	un	> 35
Rengaswamy Sankaranarayanan 2000 [25]	Asia	India	49,179	1154	un	yes	yes	un
T. Nagao 2000 [26]	Asia	Japan	19,056	37	WHO 1980	yes	yes	> 40
Dombi C 2001 [27]	Europe	Hungary	5034	167	Axe´ll T 1984	yes	no	18–89
G. Campisi 2001 [28]	Europe	Italy	118	15	WHO 1996	yes	no	≥ 40
H.C. Lin 2001 [29]	Asia	China	3088	25	WHO 1980	yes	no	35–44; 65–74
Garcı´a-Pola Vallejo MJ 2002 [30]	Europe	Spain	308	5	WHO 1996	yes	yes	> 30
Christian Scheifele 2003 [31]	North America	America	16,128	65	WHO 1978	yes	no	> 20
I. Espinoza 2003 [32]	South America	Chile	889	15	WHO 1997	yes	no	≥ 65
Paulo JoseÂ Benevides dos Santos 2004 [33]	South America	Brazil	587	1	WHO 1980	yes	no	0–45
Ching-Hung Chung 2005 [34]	Asia	China(Taiwan)	1075	80	WHO 1980	yes	no	> 15
Steven J. Thomas 2008 [35]	Oceania	Papua New Guinea	1678	197	WHO 1984	yes	no	> 18
Hemantha K.Amarasinghe 2010 [36]	Asia	Sri Lanka	1029	71	WHO 1996	yes	yes	> 30
Ravi Mehrotra 2010 [37]	Asia	India	3030	88	WHO 1980	yes	yes	un
Yi-Hsin Yang 2010 [38]	Asia	China(Taiwan)	2020	224	WHO 1980	yes	yes	≥ 35
Amy Ming-Fang Yen 2011 [39]	Asia	China(Taiwan)	79,940	368	un	yes	no	> 20
VC Carrard 2011 [40]	South America	Brazil	1586	17	WHO 1997	yes	yes	≥ 14
Fariborz Mansour Ghanaei 2013 [41]	Asia	Iran	1581	2	un	yes	yes	> 30
Sushi Kadanakuppe 2013 [42]	Asia	India	2605	3	WHO 1997	yes	no	1–80
Chang-Cheng Chang 2015 [43]	Asia	China(Taiwan)	5161	279	WHO 1997	yes	no	20–80
Elizabeth L. Yanik 2015 [44]	North America	America	470,266	1526	un	yes	un	≥ 65
José Nicolau Gheno 2015 [45]	South America	Brazil	801	11	un	yes	no	11–88
Jingqiu Feng 2015 [46]	Aisa	China	11,054	24	WHO 1980	yes	no	all
Nilesh Arjun Torwane 2015 [47]	Asia	India	432	19	un	yes	no	aver = 37.7
Divya Mehrotra 2017 [48]	Asia	India	402,669	2980	un	un	un	≥ 15
He Liang 2017 [49]	Asia	China(Mainland)	29,476	7371	WHO before 1984	yes	no	40–69
Andrej Aleksander Kansky 2018 [50]	Europe	Slovenia	2395	12	WHO 1980	yes	un	22–92
Tepirou Chher 2018 [51]	Asia	Cambodia	1298	25	un	yes	no	≥ 18
Meghashyam Bhat 2019 [52]	Asia	India	873	21	un	yes	un	35–54
2019, Sendhil Kumar [53]	Asia	India	1048	33	WHO 1980	yes	no	18–87
Carla Cruvinel Pontes 2020 [54]	Africa	South Africa	1976	48	WHO 2016	yes	no	≥ 18
Shuyun Ge 2020 [55]	Asia	China	653	1	WHO 1980	yes	yes	17–92
Ulla-Maija Oivio 2020 [56]	Europe	Finland	1961	9	WHO 1980	yes	no	44–47
Allan Vinícius Martins-de-Barros 2021 [57]	South America	Brazil	304	7	OPMD 2007	yes	no	> 40
2021, Stefan Kindler [58]	Europe	Germany	6078	40	WHO 2005	yes	no	20–79
Libin Benance Jacob 2022 [59]	Asia	India	930	29	un	yes	no	18–60

Abbreviations: *un* unclear, *aver* average

Prevalence estimates for OLK derived by meta-analysis are shown in Fig. 2. The random-effects overall pooled estimated prevalence of OLK was 2.23% (95%CI, 1.44–3.18%) with high heterogeneity (I^2^ = 99.87%; *P* < 0.0001) (Fig. 2).

In population-based studies, the overall estimated prevalence rates for Asia, Europe, South American, and North American were 2.53%, 1.82%, 1.51%, and 0.33%. The one study from Africa and Oceania showed prevalence of 2.43% and 11.74%. The inter-group heterogeneity for the different continents showed *P* < 0.001. Intra-group heterogeneity was very high (I^2^ ≥ 97.31%, *P* < 0.001) except for the heterogeneity among the studies in South America (I^2^ = 70.35%, *P* = 0.09). The heterogeneity of the studies among Asia, Europe, and South America showed no statistical differences(*P* = 0.21).

### Clinic-based studies

Table 2 lists the basic information provided in the included the clinic-based studies. All the patients visited the dentist or were from medical care/insurance. The total prevalence was 0.64% (321/50493) from 12 original studies. The random-effects pooled overall estimated prevalence of OLK was 1.36% (95% CI, 0.82–2.02%) with very high heterogeneity (I^2^ = 95.94%; *P* < 0.001). In the clinic-based studies, the overall estimated prevalence of OLK in Asia, Europe, and South America was 1.76% (9), 0.38% (2), and 0.66% (1), respectively. The overall heterogeneity of the studies from the different continents was 95.94% (*P* < 0.001).

**Table 2:** The basic information of clinic-based study

Publish year Authors	Continent	Country	Total	OLK	Definition	Examination	Biopsy	Age
A Jainkittivong 2002 [60]	Asia	Thai	500	24	WHO 1980	yes	no	≥ 60
TR Saraswathi 2006 [61]	Asia	India	2017	12	un	yes	un	13–84
Ali-Rıza-İlker Cebeci 2009 [62]	Asia	Turkey	5000	20	WHO 1980	yes	yes	17–85
Raghavendra Byakodi 2011 [63]	Asia	India	24,422	75	un	yes	yes	un
Jairo Robledo-Sierra 2013 [64]	Europe	Sweden	6448	24	WHO 1978	yes	no	21–93
Mithra N. Hegde 2014 [65]	Asia	India	2000	42	WHO 1997	yes	no	all
Queiroz 2014 [66]	South America	Brazil	6560	43	un	yes	yes	aver = 57
Y Hassona 2014 [67]	Asia	Jordan	1041	5	WHO 2005	yes	yes	16–86
Sameer Rastogi 2015 [68]	Asia	India	400	48	WHO, un	yes	no	60–100
Al-Maweri SA 2018 [69]	Asia	Yemen	1052	13	OPMD 2007	yes	yes	15–87
Christiana Madjova 2018 [70]	Europe	Bulgaria	603	6	un	yes	no	18–82
Denny E. Ceena 2022 [71]	Asia	India	450	9	un	yes	un	60–90

Abbreviations: *un* unclear, *aver* average

### Specific population studies

Table 3 lists the basic information provided in the included specific population studies. The total prevalence was 4.67% (1183/25346) from 19 original studies. The random-effects pooled overall estimated prevalence of OLK for the clinic-based studies(most patients visited the stomatology clinic) was 9.10% (95%CI, 4.85–14.47%) in 19 studies, with very high heterogeneity (I^2^ = 99.26%; *P* < 0.001). In specific population studies, the overall estimated prevalence rates of OLK in Asia, Europe, South America, North American, and Africa were 12.77% (11), 4.85% (3), 0.25% (1), 7.69% (2), and 6.26% (2), respectively. The heterogeneity of the studies between Asia and Europe showed no statistical differences (*P* = 0.14).

**Table 3:** The basic information of specific population study

Publish year Authors	Continent	Country	Total	OLK	Definition	Examination	Biopsy	Age
MAJ Jeffrey A. Grasser 1997 [72]	North America	America(Carolina)	214	5	un	yes	no	18–47
Nicola Pearson 2001 [73]	Europe	England	137	34	WHO 1980	yes	no	≥ 40
Toru Nagao 2003 [74]	Europe	England	484	16	WHO 1980	yes	no	all
Eric Oakley 2005 [75]	North America	America(sapan)	309	40	WHO 1978	yes	no	14–18
A. Ariyawardana 2007 [76]	Asia	Sri Lanka	12,716	41	WHO 1980	yes	yes	≥ 15
M Pentenero 2008 [77]	Europe	Italy	4098	47	WHO 1997	yes	yes	all
Rushabh J Dagli 2008 [78]	Asia	India	513	171	WHO 1980	yes	no	≥ 18
Reto Morger 2010 [79]	Europe	Switzerland	615	8	WHO 1980	yes	yes	18–24
Agbor MA 2013 [80]	Africa	Cameroon	226	19	un	yes	no	40–69
Santhosh Vediyera Chandroth 2014 [81]	Asia	India	979	135	WHO 2013	yes	no	≥ 18
Viviani-Silva Araújo 2015 [82]	South America	Brazil	395	1	WHO 1997	yes	un	un
Anzil KS Ali 2017 [83]	Asia	India	2163	319	un	yes	no	15–54
Punith Shetty 2017 [84]	Asia	India	450	20	un	yes	un	> 18
Nisha Rani Yadav 2018 [85]	Asia	India	464	70	un	yes	un	65–74
Shrikanth Muralidharanl 2018 [86]	Asia	India	350	110	WHO 1997	un	no	> 18
Afolabi Oyapero 2020 [87]	Afirca	Nigeria	144	5	un	yes	no	> 18
Yunus G.Y. 2021 [88]	Asia	India	185	51	un	yes	no	aver = 45
Choudhury AR 2022 [89]	Asia	India	451	31	WHO 2013	yes	un	24–60
Sandeep Kumar 2022 [90]	Asia	India	453	60	WHO 1997	yes	no	18–54

Abbreviations: *un* unclear, *aver* average

### Subgroup analysis

Table 4 lists the outcome in subgroup analysis.

- (1) The definition subgroup
  WHO issued four editions of the definition of OLK separately in 1978, 1996, 2005, and 2015. The definition in 1978 contains white lesions caused by many physical and chemical factors. In 1996, it was pointed that OLK was not associated with any physical or chemical causative agent except the use of tobacco. In 2005 and 2015, the working group emphasized the risk of OLK transforming into cancer. Among all the studies,18 were published before 2005, 51 were published after 2005 within which 24 were published after 2015. In all the 69 studies, 19 studies used the definition of OLK in 1996 and 8 of them used the definition in 2005 and after. We analyzed the prevalence rate by use of the different definitions of OLK. In population-based studies, studies using the definition from WHO in 2005 and after, 1996 and after, other definition, and unclear definition showed an estimated prevalence of 1.61%, 3.06%, 2.56%, and 1.30%, respectively. The inter-group heterogeneity showed no statistical differences (*P* > 0.05) while the intra-group heterogeneity was very high (*P* < 0.001). In the clinic-based studies and specific population, studies used the definition from WHO in 1996, other definition, and unclear definition showed an estimated prevalence without statistical differences in the inter-group heterogeneity (*P* > 0.05), while the intra-group heterogeneity was very high (*P* ≤ 0.001). (Table 4).
- (2) The sex subgroup
  In population-based studies, the overall estimated prevalence of OLK for males was 5.86% (95% CI, 1.71–12.20%) in 18 studies, and for females was 1.50% (95% CI, 0.44–3.14%) in 17 studies, without statistical differences in the inter-group heterogeneity (*P* > 0.05). In clinic-based studies, the overall estimated prevalence for males was 2.29% (95% CI, 0.88–4.31%), and for females was 1.27% (95% CI, 0.55–2.26%) in five studies, with statistical differences in the inter-group heterogeneity (*P* < 0.05). The prevalence of OLK in males was 3.9 and 1.8 times higher than that of female patients in the population and clinic-based groups, respectively. In the specific populations, the overall estimated prevalence for males was 4.69% (95% CI, 1.71–8.97%) in seven studies, and for females was 4.58% (95% CI, 1.24–9.69%) in eight studies. The heterogeneity in the specific populations between the two sex groups was no statistical difference (*P* = 0.98).
- (3) The age subgroup
  Ten studies provided age subgroup data, with two using mantissa five as the dividing line and eight using mantissa ten. Five studies divided the population into those above and below 60 years old and provide concrete data. Among the 165,496 people surveyed, 37,346 people were aged over 60 years old (22.57%). The patients over 60 years old accounted for 28.53% of the 368 patients with OLK. The ES was 2.21(95% CI, 0.09%–5.96%) over 60 years old without statistical differences in the inter-group heterogeneity (*P* = 0.21).(Table 4).
- (4) The smoking/drinking habit subgroup
  Among all the included literature, 15 studies investigated the correlation between leukoplakia and tobacco use. Among the 641,004 people surveyed, 180,898 people reported a smoking habit. The method of tobacco use comprised general (bidi, cigarette pan, pipe, and cigar smoking), reverse, and smokeless tobacco (chewing, inhalation). The population covers non-smokers, ex-smokers, and smokers who have smoked for less than five years and up to 16 years. Tobacco abuse was commonly seen in the young population (age group 15 to 34 years) [83] Regardless of the kind and frequency of tobacco use, the overall ES in population based studies was 9.48 (95% CI, 3.98–16.96) with high heterogeneity (*P* = 0.002). The overall ES in specific population was 9.59 (95% CI, 4.44–16.37) with high heterogeneity (*P* = 0.001) (Table 4).

**Table 4:** The subgroup analysis in 3 population groups

	**ES in population-based study (95%CI) %**	*P*	**ES in clinic-based study (95%CI) %**	*P*	**ES in specific population study (95%CI) %**	*P*
**Definition**		**0.35**		**0.77**		**0.95**
≥ 2005	1.61(0.41,3.52)	//	1.20(0.43,2.34)	//	8.50(1.98,18.88)	< 0.001
≥ 1996	3.06(0.91,6.35)	< 0.001				
< 1996	2.56(0.54,5.99)	< 0.001	1.14(0.30,2.50)	//	7.50(3.56,12.70)	< 0.001
un	1.30(0.88,1.79)	< 0.001	1.63(0.71,2.90)	< 0.001	9.21(1.32,22.91)	< 0.001
**Sex**		0.056		0.018		0.98
Male	5.86(1.71,12.20)	< 0.001	2.29(0.88,4.31)	< 0.001	4.69 (1.71 8.97)	< 0.001
Female	1.50(0.44,3.14)	< 0.001	1.27(0.55 2.26)	< 0.05	4.58(1.24 9.69)	< 0.001
**Continent Partial**		< 0.001 0.21		< 0.001		< 0.001 0.14
Asia	2.53(1.21,4.30)	< 0.001	1.76(0.87,2.93)	< 0.001	12.77(4.33,24.73)	< 0.001
Europe	1.82(0.63,3.55)	< 0.001	0.38(0.24,0.55)	//	4.85(1.21,10.62)	< 0.001
South America	1.15(0.59,1.88)	< 0.05	1.36(0.82,2.02)	//	0.25(0.01,1.40)	//
North America	0.33(0.31,0.34)	//	^a^	^a^	7.69(5.54,10.16)	//
Africa	2.43(1.80,3.21)	//	^a^	^a^	6.26(3.97,9.00)	//
Oceania	11.74(10.24,13.38)	//	^a^	^a^	^a^	^a^
**Age**		0.21				
≥ 60y	2.21(0.09,5.96)	< 0.001	^a^	^a^	^a^	^a^
< 60y	1.73(0.48,3.72)	< 0.001	^a^	^a^	^a^	^a^
**Smoking**		0.002				0.001
Yes	9.48(3.98,16.96)	< 0.001	^a^	^a^	9.59(4.44,16.37)	< 0.001
No	1.24(0.18,3.11)	< 0.001	^a^	^a^	1.30(0.19,3.10)	< 0.05
**Alcohol**		0.34				
Yes	10.79(1.69,26.16)	< 0.001	^a^	^a^	^a^	^a^
No	4.18(0.07,13.01)	< 0.001	^a^	^a^	^a^	^a^
**District**		0.14				^a^
Urban	1.20(0.40,2.39)	//	^a^	^a^	^a^	^a^
Rural	4.96(0.65,8.62)	< 0.001	^a^	^a^	^a^	^a^
**Bias Risk**		0.65		0.0002		0.65
Low	1.87(1.42,2.38)	< 0.001	0.45(0.30,0.62)	//	9.10(4.85,14.47)	< 0.001
Medium	2.81(0.13,8.62)	< 0.001	1.43(0.72,2.37)	< 0.001	10.09(4.68,17.22)	< 0.001
High	^a^	^a^	1.09(0.71,1.54)	//	^a^	^a^

^a^the data is absent //the size of studies is less than 4

Eight studies examined the correlation between leukoplakia and alcohol consumption. The alcohol users occupied 33.2–91.6% of the patients with OLK. Four studies only reported whether or not the population drank alcohol [34, 49, 79, 81]. Among the 165,496 people surveyed, 37,346 people have or had a drinking habit. In population studies, the ES of drinkers was 10.49 (95%CI, 1.69–26.16) without statistical differences in the inter-group heterogeneity (*P* = 0.34).

### Meta-regression, bias, and sensitivity analysis

Preliminary meta-regression analysis showed that the population could be a source of heterogeneity (*P* = 0.002), while the definition could not (*P* = 0.60). In addition, the meta-regression analysis suggested that the publication year(*P* = 0.25) and bias risk(*P* = 0.61) might not be significant contributors to the heterogeneity.

We further conducted the subgroup analysis of bias risk in 3 population sources of the studies. The result was showed in the Table 4 of the manuscript. The heterogeneity of the studies among different bias risk showed no statistical difference in population based studies(*P* = 0.65) and specific population studies(*P* = 0.65). In clinic-based studies, the heterogeneity of bias risk showed a statistical difference(*P* < 0.001). However, there are just two each for low and high-risk for clinic-based studies. The result need to be treated with caution.

We leave out 1 study at a time for sensitivity analysis(ST2). The results showed that there was no significant change in the overall heterogeneity neither intra-subgroup nor among subgroups (I^2^ > 90%, *P* < 0.05). The overall estimated prevalence in all studies ranged from 3.00%(95% CI, 2.59–3.45%) to 3.52%(95% CI, 2.59–4.58%). The overall estimated prevalence, especially the confidence interval, are relatively stable.

## Discussion

According to a global analysis in 2020, the burden of cancer incidence and mortality is growing rapidly worldwide. The number of new cases and deaths from lip and oral cancer globally were 377,713 and 177,757. Mortality ranked sixth in Southeast and West Asia and the 2022 trends in mortality rates of oral cavity and pharynx have tended to increase according to studies from the USA [91, 92]. Squamous cell carcinoma represents over 95% of oral cancer, and it can be transformed from OLK [93]. Therefore, early diagnosis and early intervention in OLK are of great importance. Epidemiological surveys based on different populations or regions are vital for precise policy on disease screening, management, prevention and living habit interventions.

In our study, the random-effects overall pooled estimated prevalence of OLK was 3.41% with high heterogeneity. The pooled estimated prevalence of the specific population studies (9.10%) was the highest among the population-based studies (2.23%) and clinic-based studies (1.36%). Research into prevalence of OPMD claimed the prevalence of OLK was 4.11% in 2018, which covered 22 studies published from 1975 to 2016 [17]. The population-base studies on the prevalence of OPMD are targeted on actinic cheilitis, oral leukoplakia, oral erythroplakia, and oral submucous fibrosis [17]. We analyzed the 69 studies published from 1996 to 2022 only in populations with OLK. The difference might have resulted from the search strategy and eligibility criteria. Our data was relatively consistent with the 2003 review who claimed the worldwide prevalence of OLK was 2.60% in studies published from 1986 to 2002 [18]. Our study showed that the prevalence of OLK is not statistically different across continents, and the global prevalence is relatively consistent. The prevalence of OLK has not changed much as the definition has been modified. The population characteristics, such as sex/age/living habits have more obvious impacts on the prevalence of OLK. The prevalence of OLK in population-based studies was 2.53% in Asia, 1.82% in Europe, 1.15% in South America, and 0.33% in North America. In clinic-based and specific population studies, the data was insufficient expect for Asia. The global prevalence of OPMD by continent was reported at 10.54% in Asia, 3.07% in Europe, 3.93% in South America and Caribbean, and 0.11% in North America [17, 94]. Difference in tobacco, betel nut, and alcohol consumption might explain some of the regional heterogeneity in the OLK global prevalence. A study published in 2020 claimed that the estimated prevalence of oral lichen planus (OLP) in population-based studies was 0.57% in Asia, 1.68% in Europe, and 1.39% in South America [95]. Asian populations had the highest prevalence in the study of OPMD and OLK, while the highest prevalence of OLP was found in in Europe. A target study for each disease included in OPMD is necessary. Considering the high heterogeneity, the outcome should be considered cautiously, and further and more widely based population-based studies might help explain this characteristic. The estimated prevalence in population based studies according to the WHO 1996 definition (3.06%) was the highest, greater than WHO 2005 definition(1.61), the other definition (2.56%) and unclear definition (1.60%). While the difference was not statistically significant (*P* = 0.34). According to the change in continent and definition, the prevalence of OLK did not show a corresponding change. Overall, the global prevalence of OLK is relatively stable across continents and definitions. However, given the limited sample size and high heterogeneity, the conclusions should be treated with caution.

Based on the presented evidence, the factors that contribute to the malignant potential of OLK include advanced age, female sex, hyperglycemia, and clinical examination characteristics [5, 96]. The factors related to the occurrence of OLK are limited and not fully consistent. A previous study on the incidence of OLK claimed that the age-adjusted incidence rate for leukoplakia was related to male sex and older age [15, 60, 97]. How the use of tobacco and alcohol affects the prevalence of OLK is controversial. The age-adjusted incidence rate for tobacco-associated leukoplakia was different in males and females [97]. Incidence of OLK was 3.22–6 times more common among smokers than among nonsmokers [97, 98]. Another study showed that the prevalence of leukoplakia among people with a smoking habit was higher, but the difference did not reach statistical significance [99]. An additional drink per day was associated with an approximately 22% increase in the risk of OLK according to a Harvard study [100]. While Nagao [99] pointed out that regular drinking was not related to the occurrence of oral leukoplakia. We found that the prevalence in males, those ≥ 60 years old, smokers, and alcohol consumers was higher, which was consistent with most previous studies. Whether these factors are related to the occurrence of OLK still requires cohort studies with a large sample of natural populations in the future. Sex, age, smoking, and alcohol consumption might be mutually confounding factors; therefore, it is more accurate to uniformly record the information in detail and analyze them separately or together to clarify the relationship between these factors and the occurrence of OLK. Besides, in human papillomavirus (HPV) infection-related regions, the prevalence of oral and pharyngeal cancer increased from 2010 to 2019 [92]. HPV infection has been suggested as a causative agent of OLK and HPV-16 has been reported to be the most prevalent HPV in OLK [25, 26, 101, 102]. However, there are few reports on this aspect in current epidemiological studies.

Our review found that the prevalence was lower in clinic-based populations than in general populations. Admission rate bias might explain the difference. Besides, other white lesions may be misdiagnosed as OLK. In 38 population-based studies, 9 studies conducted biopsy(23.68%). In 12 clinic-based studies, 5 conducted biopsy(41.67%). Both in population-based studies and clinic-based studies, the pooling prevalence were lower in biopsy studies while the heterogeneity between sub-groups showed no significant difference (*P* > 0.05). In addition, the specific population studies showed a higher prevalence than the other two groups. The specific populations cannot be simply classified into different categories by occupation, nationality, or race. The high prevalence in this group might be due to the presence of potential risk factors and selection bias, such as old age or common smoking habits. The specific population studies partly focus on the risk factors especially smoking habits. The prevalence of tobacco use was found to be 71%-90%. The proportion of OLK patient in tobacco users was reported to 13.40%-16.34% in specific population studies. Otherwise, the sample size in 11 studies is smaller than the lower quartile, sample size and sampling method probability contributed to the outcome. The outcome from the specific populations should be analyzed more rigorously because the prevalence rates varied as the population sources changed.

Several limitations of our study must be pointed out: Firstly, there was significant heterogeneity between the studies. Even though we conducted subgroup analysis, the heterogeneity within each subgroup also remained high. Secondly, detailed descriptions, such as definition, geographical location, age, sex, smoking and drinking habits, were limited, and adjusted statistics are unavailable. In addition, because of the limited number of studies, latent bias, and data heterogeneity, the interpretation of the results needs to be treated with caution.

OLK is the most common OPMD. Early diagnosis and early intervention are very important to prevent malignant transformation. Epidemiological surveys based on different populations or regions are vital to formulate precise policies on disease screening, management, prevention, and living habit interventions. Our analysis showed that the total prevalence of OLK in 63 studies published from 1996 to 2022 was 1.39%. The random-effects overall pooled estimated global prevalence was 3.41%. The pooled prevalence in different continents ranged from 0.33% to 11.74% without a statistical difference in the population-based studies.

Future studies are warranted to assess the prevalence accurately, to assess the clinical and financial burden of OLK worldwide, and test new strategies for OLK prevention and control, especially in populations with a high prevalence of OLK.

## Conclusion

The total prevalence of OLK was 1.39%, and the random-effects overall pooled estimated global prevalence was 3.41%. The overall pooled estimated prevalence of OLK among different population source of studies shows a statistically significant difference. The pooled prevalence in different continents ranged from 0.33 to 11.74% in population-based studies. The prevalence of OLK was relatively consistent and stable across different continents and different definitions. The pooled estimated prevalence of males was higher than in females, with statistically significant differences in clinic-based studies. A higher pooled estimated prevalence was found among people aged over 60 years old, the smoking population, and those consuming alcohol. Certain special populations suffer from higher rates of OLK. More study is required to develop early treatment and clinical surveillance strategies, as well as to effect habit intervention in these populations.

### Supplementary Information

**Additional file 1.** 

**Additional file 2.** 

**Additional file 3.** 

**Additional file 4.**

## Data Availability

All data generated or analysed during this study are included in this manuscript and its supplementary information files.

## References

[1] Warnakulasuriya S, Kujan O, Aguirre-Urizar JM, Bagan JV, González-Moles MÁ, Kerr AR (2021). Oral potentially malignant disorders: a consensus report from an international seminar on nomenclature and classification, convened by the WHO Collaborating Centre for Oral Cancer. Oral Dis.

[2] Van Der Meij EH, De Visscher JGAM. Leukoplakia of the oral mucosa, Nederlands Tijdschrift voor Dermatologie en Venereologie. 2017;27(4):186–8.

[3] Aguirre-Urizar JM, Lafuente-Ibáñez de Mendoza I, Warnakulasuriya S. Malignant transformation of oral leukoplakia: systematic review and meta-analysis of the last 5 years. Oral Dis. 2021;27(8):1881–95. 10.1111/odi.13810. 10.1111/odi.13810 33606345

[4] Warnakulasuriya S, Ariyawardana A (2016). Malignant transformation of oral leukoplakia: a systematic review of observational studies. J Oral Pathol Med.

[5] Evren I, Brouns ER, Wils LJ, Poell JB, Peeters C, Brakenhoff RH, et al. Annual malignant transformation rate of oral leukoplakia remains consistent: A long-term follow-up study. Oral Oncol. 2020;110:105014. 10.1016/j.oraloncology.2020.105014 10.1016/j.oraloncology.2020.105014 33038723

[6] Iocca O, Sollecito TP, Alawi F, Weinstein GS, Newman JG, De Virgilio A (2020). Potentially malignant disorders of the oral cavity and oral dysplasia: a systematic review and meta-analysis of malignant transformation rate by subtype. Head Neck.

[7] Zanoni DK, Montero PH, Migliacci JC, Shah JP, Wong RJ, Ganly I (2019). Survival outcomes after treatment of cancer of the oral cavity (1985–2015). Oral Oncol.

[8] Jäwert F, Nyman J, Olsson E, Adok C, Helmersson M, Öhman J (2021). Regular clinical follow-up of oral potentially malignant disorders results in improved survival for patients who develop oral cancer. Oral Oncol.

[9] Bouaoud J, Bossi P, Elkabets M, Schmitz S, van Kempen LC, Martinez P, et al. Unmet needs and perspectives in oral cancer prevention. Cancers (Basel). 2022;14(7):1815. 10.3390/cancers14071815. 10.3390/cancers14071815 PMC8997728 35406587

[10] Kramer IR, Lucas RB, Pindborg JJ, Sobin LH (1978). Definition of leukoplakia and related lesions: an aid to studies on oral precancer. Oral Surg Oral Med Oral Pathol.

[11] Axéll T, Holmstrup P, Kramer IRH, Pindborg JJ, Shear M (1984). International seminar on oral leukoplakia and associated lesions related to tobacco habits. Commun Dent Oral Epidemiol.

[12] Axéll T, Pindborg JJ, Smith CJ, Waal I (1996). Oral white lesions with special reference to precancerous and tobacco- related lesions: conclusions of an international symposium held in Uppsala, Sweden, May 18–21 1994. International Collaborative Group on Oral White Lesions. J Oral Pathol Med.

[13] van der Waal I. Oral leukoplakia, the ongoing discussion on definition and terminology. Med Oral Patol Oral Cir Bucal. 2015;20(6):e685–92. 10.4317/medoral.21007. 10.4317/medoral.21007 PMC4670248 26449439

[14] Warnakulasuriya S, Johnson NW, van der Waal I (2007). Nomenclature and classification of potentially malignant disorders of the oral mucosa. J Oral Pathol Med.

[15] Maymone MBC, Greer RO, Kesecker J (2019). Premalignant and malignant oral mucosal lesions: clinical and pathological findings. J Am Acad Dermatol.

[16] Pritzker KPH, Darling MR, Hwang JTK, Mock D (2021). Oral Potentially Malignant Disorders (OPMD): What is the clinical utility of dysplasia grade?. Expert Rev Mol Diagn.

[17] Mello FW, Miguel AFP, Dutra KL, Porporatti AL, Warnakulasuriya S, Guerra ENS (2018). Prevalence of oral potentially malignant disorders: a systematic review and meta-analysis. J Oral Pathol Med.

[18] Petti S (2003). Pooled estimate of world leukoplakia prevalence: a systematic review. Oral Oncol.

[19] Roza ALOC, Kowalski LP, William WN Jr, de Castro G Jr, Chaves ALF, Araújo ALD, et al. Oral leukoplakia and erythroplakia in young patients: a systematic review. Oral Surg Oral Med Oral Pathol Oral Radiol. 2021;131(1):73-84. 10.1016/j.oooo.2020.09.002 10.1016/j.oooo.2020.09.002 33187936

[20] Shamseer L, Moher D, Clarke M, Ghersi D, Liberati A, Petticrew M, et al. Preferred reporting items for systematic review and meta-analysis protocols (PRISMA-P) 2015: elaboration and explanation [published correction appears in BMJ. 2016;354:i4086]. BMJ. 2015;350:g7647. 10.1136/bmj.g7647 10.1136/bmj.g7647 25555855

[21] Munn Z, Moola S, Lisy K, Riitano D, Tufanaru C (2015). Methodological guidance for systematic reviews of observational epidemiological studies reporting prevalence and cumulative incidence data. Int J Evid Based Healthc.

[22] Ali TB, Jalalluddin RL, Abdul Razak I, Zain RB. Prevalence of oral precancerous and cancerous lesions in elderly Malaysians. Asia Pac J Public Health. 1996;9:24-27. 10.1177/101053959700900105. 10.1177/101053959700900105 10050195

[23] Zain RB, Ikeda N, Razak IA, Axell T, Majid ZA, Gupta PC, et al. A national epidemiological survey of oral mucosal lesions in Malaysia. Community Dentistry and Oral Epidemiology. 1997;25:377-83. 10.1111/j.1600-0528.1997.tb00959.x. 10.1111/j.1600-0528.1997.tb00959.x 9355776

[24] Hashibe M, Sankaranarayanan R, Thomas G, Kuruvilla B, Mathew B, Somanathan T, et al. Alcohol drinking, body mass index and the risk of oral leukoplakia in an Indian population. International Journal of Cancer. 2000;88:129-34. 10.1002/1097-0215(20001001)88:1<129::AID-IJC20>3.0.CO;2-U. 10962450

[25] Sankaranarayanan R, Mathew B, Jacob BJ, Thomas G, Somanathan T, Pisani P, et al. Early findings from a community-based, cluster-randomized, controlled oral cancer screening trial in Kerala, India. Cancer. 2000;88:664-73.10.1002/(SICI)1097-0142(20000201)88:3<664::AID-CNCR25>3.0.CO;2-V. 10649262

[26] Nagao T, Ikeda N, Fukano H, Miyazaki H, Yano M, Warnakulasuriya S. Outcome following a population screening programme for oral cancer and precancer in Japan. Oral Oncology. 2000;36:340-6. 10.1016/S1368-8375(00)00011-7. 10.1016/s1368-8375(00)00011-7 10899672

[27] Dombi C, Voros-Balog T, Czegledy A, Hermann P, Vincze N, Banoczy J. Risk group assessment of oral precancer attached to X-ray lung-screening examinations. Community Dentistry and Oral Epidemiology. 2001;29:9-13. 11153567

[28] Campisi G, Margiotta V. Oral mucosal lesions and risk habits among men in an Italian study population. Journal of Oral Pathology and Medicine. 2001;30:22-8. 10.1034/j.1600-0714.2001.300104.x. 10.1034/j.1600-0714.2001.300104.x 11140896

[29] Lin HC, Corbet EF, Lo ECM. Oral mucosal lesions in adult Chinese. Journal of Dental Res. 2001;80(5):1486-90. 10.1177/00220345010800052001. 10.1177/00220345010800052001 11437225

[30] García-Pola Vallejo MJ, Martínez Díaz-Canel AI, García Martín JM, González García M. Risk factors for oral soft tissue lesions in an adult Spanish population. Community Dent Oral Epidemiol. 2002;30(4):277-85. 10.1034/j.1600-0528.2002.00048.x. 10.1034/j.1600-0528.2002.00048.x 12147169

[31] Scheifele C, Reichart PA, Dietrich T. Low prevalence of oral leukoplakia in a representative sample of the US population. Oral Oncology. 2003;39:619-25. 10.1016/S1368-8375(03)00050-2. 10.1016/s1368-8375(03)00050-2 12798406

[32] Espinoza I, Rojas R, Aranda W, Gamonal J. Prevalence of oral mucosal lesions in elderly people in Santiago, Chile. Journal of Oral Pathology and Medicine. 2003;32:571-5. 10.1034/j.1600-0714.2003.00031.x. 10.1034/j.1600-0714.2003.00031.x 14632931

[33] Benevides Dos Santos PJ, Navarro Bessa CF, Ferreira De Aguiar MC, Vieira Do Carmo MA. Cross-sectional study of oral mucosal conditions among a central Amazonian Indian community, Brazil. Journal of Oral Pathology and Medicine. 2004;33:7-12. 10.1111/j.1600-0714.2004.00003.x. 10.1111/j.1600-0714.2004.00003.x 14675134

[34] Chung CH, Yang YH, Wang TY, Shieh TY, Warnakulasuriya S. Oral precancerous disorders associated with areca quid chewing, smoking, and alcohol drinking in southern Taiwan. Journal of Oral Pathology and Medicine. 2005;34:460–6. 10.1111/j.1600-0714.2005.00332.x. 10.1111/j.1600-0714.2005.00332.x 16091112

[35] Thomas SJ, Harris R, Ness AR, Taulo J, Maclennan R, Howes N, et al. Betel quid not containing tobacco and oral leukoplakia: A report on a cross-sectional study in Papua New Guinea and a meta-analysis of current evidence. International Journal of Cancer. 2008;123:1871-6. 10.1002/ijc.23739. 10.1002/ijc.23739 18688850

[36] Amarasinghe HK, Usgodaarachchi US, Johnson NW, Lalloo R, Warnakulasuriya S. Betel-quid chewing with or without tobacco is a major risk factor for oral potentially malignant disorders in Sri Lanka: A case-control study. Oral Oncology. 2010;46:297-301. 10.1016/j.oraloncology.2010.01.017. 10.1016/j.oraloncology.2010.01.017 20189448

[37] Mehrotra R, Thomas S, Nair P, Pandya S, Singh M, Nigam NS, et al. Prevalence of oral soft tissue lesions in Vidisha. BMC Research Notes. 2010;3:23. 10.1186/1756-0500-3-23. 10.1186/1756-0500-3-23 PMC2828461 20181008

[38] Yang YH, Ho PS, Lu HM, Huang IY, Chen CH. Comparing dose-response measurements of oral habits on oral leukoplakia and oral submucous fibrosis from a community screening program. Journal of Oral Pathology and Medicine. 2010;39:306-12. 10.1111/j.1600-0714.2009.00820.x 10.1111/j.1600-0714.2009.00820.x 20149061

[39] Yen AM, Chen SL, Chiu SY, Chen HH. Association between metabolic syndrome and oral pre-malignancy: a community- and population-based study (KCIS No. 28). Oral oncology. 2011;47:625-30. 10.1016/j.oraloncology.2011.04.011. 10.1016/j.oraloncology.2011.04.011 21592847

[40] Carrard V, Haas A, Rados P, Filho M, Oppermann R, Albandar J, et al. Prevalence and risk indicators of oral mucosal lesions in an urban population from South Brazil. Oral diseases. 2011;17:171-9. 10.1111/j.1601-0825.2010.01712.x. 10.1111/j.1601-0825.2010.01712.x 20659262

[41] Ghanaei FM, Joukar F, Rabiei M, Dadashzadeh A, Valeshabad AK. Prevalence of Oral Mucosal Lesions in an Adult Iranian Population. Iranian Red Crescent Medical Journal. 2013;15:600-4. 10.5812/ircmj.4608. 10.5812/ircmj.4608 PMC3871749 24396581

[42] Kadanakuppe S, Bhat PK. Oral health status and treatment needs of Iruligas at Ramanagara District, Karnataka, India. The West Indian medical journal. 2013;62:73-80. 10.7727/wimj.2012.189. 24171332

[43] Chang CC, Lin MS, Chen YT, Tu LT, Jane SW, Chen MY. Metabolic syndrome and health-related behaviours associated with pre-oral cancerous lesions among adults aged 20-80 years in Yunlin County, Taiwan: a cross-sectional study. BMJ open. 2015;5:e008788. 10.1136/bmjopen-2015-008788. 10.1136/bmjopen-2015-008788 PMC4691787 26685025

[44] Yanik EL, Katki HA, Silverberg MJ, Manos MM, Engels EA, Chaturvedi AK. Leukoplakia, oral cavity cancer risk, and cancer survival in the U.S. elderly. Cancer Prevention Research. 2015;8:857-63. 10.1158/1940-6207.CAPR-15-0091. 10.1158/1940-6207.CAPR-15-0091 PMC4560597 26159805

[45] Gheno JN, Trevizani Martins MA, Munerato MC, Hugo FN, Sant'Ana Filho M, Weissheimer C, et al. Oral mucosal lesions and their association with sociodemographic, behavioral, and health status factors. Brazilian Oral Research. 2015;29. 10.1590/1807-3107BOR-2015.vol29.0093. 10.1590/1807-3107BOR-2015.vol29.0093 26247518

[46] Feng J, Zhou Z, Shen X, Wang Y, Shi L, Wang Y, et al. Prevalence and distribution of oral mucosal lesions: A cross-sectional study in Shanghai, China. Journal of Oral Pathology and Medicine. 2015;44:490-4. 10.1111/jop.12264. 10.1111/jop.12264 25243724

[47] Torwane NA, Hongal S, Goel P, Chandrashekar B, Saxena V. Assessment of oral mucosal lesions among eunuchs residing in Bhopal city, Madhya Pradesh, India: a cross-sectional study. Indian journal of public health. 2015;59:24-9. 10.4103/0019-557X.152851. 10.4103/0019-557X.152851 25758727

[48] Mehrotra D, Kumar S, Mishra S, Kumar S, Mathur P, Pandey CM, et al. Pan masala habits and risk of oral precancer: A cross-sectional survey in 0.45 million people of North India. Journal of oral biology and craniofacial research. 2017;7:13-8. 10.1016/j.jobcr.2016.12.003. 10.1016/j.jobcr.2016.12.003 PMC5343152 28316915

[49] Liang H, Yang Z, Wang JB, Yu P, Fan JH, Qiao YL, et al. Association between oral leukoplakia and risk of upper gastrointestinal cancer death: A follow-up study of the Linxian General Population Trial. Thoracic cancer. 2017;8:642-8. 10.1111/1759-7714.12501. 10.1111/1759-7714.12501 PMC5707438 28929584

[50] Kansky AA, Didanovic V, Dovsak T, Brzak BL, Pelivan I, Terlevic D. Epidemiology of oral mucosal lesions in Slovenia. Radiology and Oncology. 2018;52:263-6. 10.2478/raon-2018-0031. 10.2478/raon-2018-0031 PMC6137360 30210036

[51] Chher T, Hak S, Kallarakkal TG, Durward C, Ramanathan A, Ghani WMN, et al. Prevalence of oral cancer, oral potentially malignant disorders and other oral mucosal lesions in Cambodia. Ethnicity & health. 2018;23:1-15. 10.1080/13557858.2016.1246431. 10.1080/13557858.2016.1246431 27781495

[52] Bhat M, Bhat S, Roberts-Thomson K, Do LG. Is Periodontitis Independently Associated with Potentially Malignant Disorders of the Oral Cavity? Asian Pacific journal of cancer prevention: APJCP. 2019;20:283-7. 10.31557/APJCP.2019.20.1.283. 10.31557/APJCP.2019.20.1.283 PMC6485564 30678451

[53] Kumar S, Narayanan VS, Ananda SR, Kavitha AP, Krupashankar R. Prevalence and risk indicators of oral mucosal lesions in adult population visiting primary health centers and community health centers in Kodagu district. Journal of family medicine and primary care. 2019;8:2337-42. 10.4103/jfmpc.jfmpc_344_19 10.4103/jfmpc.jfmpc_344_19 PMC6691453 31463253

[54] Pontes CC, Chikte U, Kimmie-dhansay F, Erasmus RT, Kengne AP, Matsha TE. Prevalence of oral mucosal lesions and relation to serum cotinine levels—findings from a cross- sectional study in South Africa. International Journal of Environmental Research and Public Health. 2020;17. 10.3390/ijerph17031065. 10.3390/ijerph17031065 PMC7037025 32046216

[55] Ge S, Liu L, Zhou Q, Lou B, Zhou Z, Lou J, et al. Prevalence of and related risk factors in oral mucosa diseases among residents in the Baoshan District of Shanghai, China. PeerJ. 2020;2020. 10.7717/peerj.8644. 10.7717/peerj.8644 PMC7045885 32140308

[56] Oivio UM, Pesonen P, Ylipalosaari M, Kullaa A, Salo T. Prevalence of oral mucosal normal variations and lesions in a middle-aged population: a Northern Finland Birth Cohort 1966 study. BMC oral health. 2020;20:357. 10.1186/s12903-020-01351-9. 10.1186/s12903-020-01351-9 PMC7727189 33298037

[57] Martins-De-barros AV, Barros AMI, Silva CCG, Ramos LFS, Ferreira SJ, Da Costa Araújo FA, et al. High prevalence of oral potentially malignant disorders and risk factors in a semi-urban Brazilian city: A population-based cross-sectional study. Medicina Oral Patologia Oral y Cirugia Bucal. 2021;26:e778-e85. 10.4317/medoral.24747. 10.4317/medoral.24747 PMC8601640 34023843

[58] Kindler S, Samietz S, Dickel S, Mksoud M, Kocher T, Lucas C, et al. Prevalence and risk factors of potentially malignant disorders of the mucosa in the general population Mucosa lesions a general health problem? Annals of Anatomy-Anatomischer Anzeiger. 2021;237. 10.1016/j.aanat.2021.151724. 10.1016/j.aanat.2021.151724 33798694

[59] Jacob LB, Jesija JS, Mohan M, Pricilla RA, Prasad JH. Prevalence of oral lesions and nicotine dependency among tobacco users in an urban community of Vellore, South India. J Clin Diagnostic Res. 2022;16:ZC31–7. 10.7860/JCDR/2022/51308.16156.

[60] Jainkittivong A, Aneksuk V, Langlais RP (2002). Oral mucosal conditions in elderly dental patients. Oral Dis.

[61] Saraswathi T, Ranganathan K, Shanmugam S, Sowmya R, Narasimhan P, Gunaseelan R (2006). Prevalence of oral lesions in relation to habits: Cross-sectional study in South India. Indian J Dent Res.

[62] Cebeci ARI, Gülşahi A, Kamburoǧlu K, Orhan BK, Öztaş B (2009). Prevalence and distribution of oral mucosal lesions in an adult turkish population. Medicina Oral, Patologia Oral y Cirugia Bucal.

[63] Byakodi R, Shipurkar A, Byakodi S, Marathe K. Prevalence of oral soft tissue lesions in Sangli, India. Journal of community health. 2011;36:756-9. 10.1007/s10900-011-9370-x. 10.1007/s10900-011-9370-x 21318256

[64] Robledo-Sierra J, Mattsson U, Svedensten T, Jontell M. The morbidity of oral mucosal lesions in an adult Swedish population. Medicina Oral Patologia Oral Y Cirugia Bucal. 2013;18:E766–72. 10.4317/medoral.19286. 10.4317/medoral.19286 PMC3790650 23792308

[65] Hegde MN, Jain R, Punja A (2014). Prevalence of oral mucosal lesions and their co - relation to habits in patients visiting a Dental School of South Karnataka: a cross sectional survey- 2012. Nitte University Journal of Health Science.

[66] Queiroz SIML, De Medeiros AMC, Silva JSPD, Silveira EJDD (2014). Clinical and histopathological evaluation and habits associated with the onset of oral leukoplakia and erythroplakia. Jornal Brasileiro de Patologia e Medicina Laboratorial.

[67] Hassona Y, Scully C, Almangush A, Baqain Z, Sawair F (2014). Oral potentially malignant disorders among dental patients: a pilot study in Jordan. Asian Pacific journal of cancer prevention : APJCP.

[68] Rastogi S, Arora P, Kapoor S, Wazir S, Vashishth S, Sharma V (2015). Prevalence of oral soft tissue lesions and medical assessment of geriatric outpatients in North India. Journal of Indian Academy of Oral Medicine and Radiology.

[69] Al-Maweri SA, Al-Jamaei A, Saini R, Laronde DM, Sharhan A. White oral mucosal lesions among the Yemeni population and their relation to local oral habits. J Investig Clin Dent. 2018;9:e12305. 10.1111/jicd.12305. 10.1111/jicd.12305 29178288

[70] Madjova C, Chokanov S (2018). Oral and dental status of bulgarian patients - a 5-year study. Journal of Imab.

[71] Ceena DE, Navya K, Nayak SU, Shenoy R, Binnal A, Bastian TS. Oral health status among the geriatric population - a cross sectional study. J Gerontology Geriatrics. 2022;70:164–8. 10.36150/2499-6564-N477.

[72] Grasser JA, Childers E (1997). Prevalence of smokeless tobacco use and clinical oral leukoplakia in a military population. Mil Med.

[73] Pearson N, Croucher R, Marcenes W, O'Farrell M (2001). Prevalence of oral lesions among a sample of Bangladeshi medical users aged 40 years and over living in Tower Hamlets, UK. Int Dent J.

[74] Nagao T, Warnakulasuriya S, Gelbier S, Yuasa H, Tsuboi S, Nakagaki H (2003). Oral pre-cancer and the associated risk factors among industrial workers in Japan's overseas enterprises in the UK. J Oral Pathol Med.

[75] Oakley E, Demaine L, Warnakulasuriya S (2005). Areca (betel) nut chewing habit among high-school children in the Commonwealth of the Northern Mariana Islands (Micronesia). Bull World Health Organ.

[76] Ariyawardana A, Sitheeque MAM, Ranasinghe AW, Perera I, Tilakaratne WM, Amaratunga EAPD (2007). Prevalence of oral cancer and pre-cancer and associated risk factors among tea estate workers in the central Sri Lanka. J Oral Pathol Med.

[77] Pentenero M, Broccoletti R, Carbone M, Conrotto D, Gandolfo S (2008). The prevalence of oral mucosal lesions in adults from the Turin area. Oral Dis.

[78] Dagli RJ, Kumar S, Mathur A, Balasubrimanyam G, Duraiswamy P, Kulkarni S. Prevalence of leukoplakia, oral submucous fibrosis, papilloma and its relation with stress among green marbles mine laborers, India. Medicina Oral, Patologia Oral y Cirugia Bucal. 20. 18978707

[79] Morger R, Ramseier CA, Rees TD, Bürgin WB, Bornstein MM (2010). Oral mucosal findings related to tobacco use and alcohol consumption: a study on Swiss army recruits involving self-reported and clinical data. Oral Health Prev Dent.

[80] Agbor MA, Azodo CC, Tefouet TSM (2013). Smokeless tobacco use, tooth loss and oral health issues among adults in Cameroon. Afr Health Sci.

[81] Chandroth SV, Venugopal HK, Puthenveetil S, Jayaram A, Mathews J, Suresh N (2014). Prevalence of oral mucosal lesions among fishermen of Kutch coast, Gujarat. India Int Marit Health.

[82] Araujo VS, Godinho EL, Farias LC, Marques-Silva L, Santos SHS, Rodrigues-Neto JF, et al. Prevalence of oral mucosal lesions in a brazilian military police population. J Clin Exp Dent. 2015;7:e208-11. 10.4317/jced.51934. 10.4317/jced.51934 PMC4483325 26155334

[83] Ali AK, Mohammed A, Thomas AA, Paul S, Shahul M, Kasim K. Tobacco abuse and associated oral lesions among interstate migrant construction workers. J Contemp Dent Pract. 2017;18:695–9. 10.5005/jp-journals-10024-2109 28816192

[84] Shetty P, Khargekar NC, Debnath A, Khargekar NR, Srivastava BK, Hakeen NEF. Determinants of tobacco use and prevalence of oral precancerous lesions in cab drivers in Bengaluru City, India. Int J Prev Med. 2017;8. 10.4103/ijpvm.IJPVM_225_17 10.4103/ijpvm.IJPVM_225_17 PMC5738789 29291042

[85] Yadav NR, Jain M, Sharma A, Yadav R, Pahuja M, Jain V (2018). Distribution and prevalence of oral mucosal lesions in residents of old age homes in Delhi, India. Nepal J Epidemiol.

[86] Muralidharan S, Acharya A, Sevekari T, Wadwan S, Joglekar NR, Margabandhu S (2018). Prevalence of soft-tissue lesions among women in sex work in the red light Area of Pune, India: a cross-sectional survey. Journal of International Society of Preventive & Community Dentistry.

[87] Oyapero A, Oyapero O, Akinleye AI. Burden of tobacco, kola nut and alcohol consumption and its association with periodontal disease, potentially malignant lesions and quality of life among bus drivers, Lagos State, Nigeria. Population Med. 2020;2. 10.18332/popmed/118726.

[88] Yunus GY, Sahni H, Naveen N, Tiwari R, Vasant B, Suman S (2019). "Tobacco" - The Silent Slayer for Oral Premalignant Lesions/Conditions among Beedi Rolling Workers of Durg City, Chhattisgarh, India: A Cross-Sectional Study. J Indian Assoc Public Health Dent.

[89] Choudhury AR, Ankola AV, Roopali S, Siddibhavi M, Vallakunja D, Khot AP, et al. Assessment of oral health status and tobacco-related habits among the employees of North-West Karnataka Road Transport Corporation (NWKRTC), Belagavi City, India - A Cross-Sectional Study. Int J Occup Saf Health. 2022;12:299–306. 10.3126/ijosh.v12i4.43885.

[90] Kumar S, Priyaranjan P, Basak D, Dasgupta B, Nastaran Quazi S, Kumar A. Oral health status and treatment needs of chromium mine workers in India. Indian Journal of Occupational and Environmental Medicine. 2022;26:172–7. 10.4103/ijoem.ijoem_223_21. 10.4103/ijoem.ijoem_223_21 PMC9674078 36408426

[91] Sung H, Ferlay J, Siegel RL, Laversanne M, Soerjomataram I, Jemal A (2021). Global Cancer Statistics 2020: GLOBOCAN Estimates of Incidence and Mortality Worldwide for 36 Cancers in 185 Countries. CA Cancer J Clin.

[92] Siegel RL, Miller KD, Fuchs HE, Jemal A (2022). Cancer statistics, 2022. CA Cancer J Clin.

[93] Bhattacharya A, Roy R, Snijders AM, Hamilton G, Paquette J, Tokuyasu T (2011). Two distinct routes to oral cancer differing in genome instability and risk for cervical node metastasis. Clin Cancer Res.

[94] Richards D (2018). Prevalence of oral potentially malignant disorders. Evid Based Dent.

[95] Li C, Tang X, Zheng X, Ge S, Wen H, Lin X (2020). Global prevalence and incidence estimates of oral lichen planus: a systematic review and meta-analysis. JAMA Dermatol.

[96] Li J, Liu Y, Zhang H, Hua H (2020). Association between hyperglycemia and the malignant transformation of oral leukoplakia in China. Oral Dis.

[97] Warnakulasuriya S (2018). Clinical features and presentation of oral potentially malignant disorders. Oral Surg Oral Med Oral Pathol Oral Radiol.

[98] Shiu MN, Chen TH, Chang SH, Hahn LJ. Risk factors for leukoplakia and malignant transformation to oral carcinoma: a leukoplakia cohort in Taiwan. Br J Cancer. 2000;82(11):1871-4. 10.1054/bjoc.2000.1208. 10.1054/bjoc.2000.1208 PMC2363234 10839305

[99] Nagao T, Ikeda N, Fukano H, Hashimoto S, Shimozato K, Warnakulasuriya S. Incidence rates for oral leukoplakia and lichen planus in a Japanese population. J Oral Pathol Med. 2005;34(9):532-9. 10.1111/j.1600-0714.2005.00349.x. 10.1111/j.1600-0714.2005.00349.x 16138891

[100] Maserejian NN, Joshipura KJ, Rosner BA, Giovannucci E, Zavras AI. Prospective study of alcohol consumption and risk of oral premalignant lesions in men. Cancer Epidemiology Biomarkers & Prevention. 2006;15:774-81. 10.1158/1055-9965.Epi-05-0842. 10.1158/1055-9965.EPI-05-0842 16614123

[101] Sundberg J, Öhman J, Korytowska M, Wallström M, Kjeller G, Andersson M, et al. High-risk human papillomavirus in patients with oral leukoplakia and oral squamous cell carcinoma-A multi-centre study in Sweden, Brazil and Romania. Oral Dis. 2021;27(2):183-92. 10.1111/odi.13510. 10.1111/odi.13510 32568421

[103] Feller L, Lemmer J. Oral leukoplakia as it relates to HPV infection: A review. International Journal of Dentistry. 2012;1-7. 10.1155/2012/540561. 10.1155/2012/540561 PMC3299253 22505902

