# Supplementary material for: The global prevalence of oral leukoplakia: a systematic review and meta-analysis from 1996 to 2022
Source: BMC Oral Health. 2023 Sep 6;23:645. doi: 10.1186/s12903-023-03342-y (PMC10481497; doi:10.1186/s12903-023-03342-y)
Supplement: Supplementary file 2 — Additional file 2. [file 12903_2023_3342_MOESM2_ESM.docx]

**Supplement 1**

**Search strategy**

*Embase:* ('oral leukoplakia':ti,ab,kw OR olk:ti,ab,kw OR leukoplakia:ti,ab,kw) AND 'oral leukoplakia':ti,ab,kw AND [1996-2022]/py

*Pubmed:* (("leukoplakia, oral"[MeSH Terms] OR ("leukoplakia"[All Fields] AND "oral"[All Fields]) OR "oral leukoplakia"[All Fields] OR ("oral"[All Fields] AND "leukoplakia"[All Fields]) OR "OLK"[All Fields] OR ("leucoplakias"[All Fields] OR "leukoplakia"[MeSH Terms] OR "leukoplakia"[All Fields] OR "leucoplakia"[All Fields] OR "leukoplakias"[All Fields])) AND ("epidemiology"[MeSH Subheading] OR "epidemiology"[All Fields] OR "prevalence"[All Fields] OR "prevalence"[MeSH Terms] OR "prevalance"[All Fields] OR "prevalences"[All Fields] OR "prevalence s"[All Fields] OR "prevalent"[All Fields] OR "prevalently"[All Fields] OR "prevalents"[All Fields] OR ("epidemiology"[MeSH Subheading] OR "epidemiology"[All Fields] OR "incidence"[All Fields] OR "incidence"[MeSH Terms] OR "incidences"[All Fields] OR "incident"[All Fields] OR "incidents"[All Fields]) OR ("epidemiologies"[All Fields] OR "epidemiology"[MeSH Subheading] OR "epidemiology"[All Fields] OR "epidemiology"[MeSH Terms] OR "epidemiology s"[All Fields]))) AND (1996:2022[pdat])

## *Scopus:* ( TITLE-ABS-KEY ( oral  AND leukoplakia  OR  olk  OR  leukoplakia )  AND  TITLE-ABS-KEY ( prevalence  OR  incidence  OR  epidemiology ) )  AND  PUBYEAR  >  1995  AND  PUBYEAR  <  2023  AND  PUBYEAR  >  1995  AND  PUBYEAR  <  2023

*Web of science:* (oral leukoplakia OR OLK OR leukoplakia (Topic) and prevalence OR incidence OR epidemiology (Topic)) AND(1996-01-01 to 2022-12-31 (Publication Date))

**Analysis outcome**

1.Population-based study

Supplementary Figure 1 Subgroup analysis by sex

Supplementary Figure 2 Subgroup analysis by continent

Supplementary Figure 3 Subgroup analysis by continent（India）

Supplementary Figure 4 Subgroup analysis by definition

2.Clinic-based population study

Supplementary Figure 5 Subgroup analysis by sex in clinic-based population studies

Supplementary Figure 6 Subgroup analysis by continent in clinic-based population studies

Supplementary Figure 7 Subgroup analysis by definition in clinic-based population studies

3.Specific population study

Supplementary Figure 8 Subgroup analysis by sex in specific population studies

Supplementary Figure 9 Subgroup analysis by continent in specific population studies

Supplementary Figure 10 Subgroup analysis by definition in specific population studies

4.Subgroup analysis by risk factors

Supplementary Figure 11 Subgroup analysis by smoking habits in specific population

Supplementary Figure 12 Subgroup analysis by smoking habits in population-based studies

Supplementary Figure 13 Subgroup analysis by drinking habits in population-based studies

Supplementary Figure 14 Subgroup analysis by age in population-based studies

Supplementary Supplementary Figure 15 Subgroup analysis by area in population-based studies
