# Supplementary material for: The global prevalence of oral leukoplakia: a systematic review and meta-analysis from 1996 to 2022
Source: BMC Oral Health. 2023 Sep 6;23:645. doi: 10.1186/s12903-023-03342-y (PMC10481497; doi:10.1186/s12903-023-03342-y)
Supplement: Supplementary file 3 — Additional file 3. [file 12903_2023_3342_MOESM3_ESM.docx]

**Supplement 2**

**Heterogeneity Analysis**

| Domain evaluated | Risk of bias | Characterization of risk of bias in analyzed studies |
| --- | --- | --- |
| *Q1* | 55.1% | Was the sample frame appropriate to address the target population?  Population-based  Clinic-based  Specific |
|  | 17.4% |  |
|  | 27.5% |  |
| *Q2* | 65.2% | Were study participants sampled in an appropriate way?  Yes  No  Un |
|  | 17.4% |  |
|  | 17.4% |  |
| *Q3* | 24.6% | Was the sample size adequate?  Sample size>=5017  458.5<=Sample size<5017  Sample size<458.5 |
|  | 50.7% |  |
|  | 24.6% |  |
| *Q4* | 79.7% | Were the study subjects and the setting described in detail?  Yes(When, Where)  No  Un |
|  | 20.3% |  |
|  | 0% |  |
| *Q5* | 68.1% | Was the data analysis conducted with sufficient coverage of the identified sample?  Yes  No  Un |
|  | 17.4% |  |
|  | 14.5% |  |
| *Q6* | 29.0% | Was the diagnostic criteria clearly elaborated？/ Were valid methods used for the identfication of the condition?  Yes(WHO 1996)  No  Un |
|  | 39.1% |  |
|  | 31.9% |  |
| *Q7* | 87.0% | Was the condition measured in a standard, reliable way for all participants?  Yes  Partial description  No |
|  | 5.8% |  |
|  | 5.8% |  |
| *Q8* | 82.6% | Was there appropriate statistical analysis?  Yes(Age, Sex)  No  Un |
|  | 15.9% |  |
|  | 1.4% |  |
| *Q9* | 43.5% | Was the response rate adequate, and if not(<70%), was the low response rate managed appropriately?  Yes  No  Un |
|  | 13.0% |  |
|  | 29.0% |  |

| Publish year+Authors（3） | Q1 | Q2 | Q3 | Q4 | Q5 | Q6 | Q7 | Q8 | Q9 |
| --- | --- | --- | --- | --- | --- | --- | --- | --- | --- |
| 1996，Tara B Taiyeb Ali | yes | yes | no | no | yes | no | yes | yes | un |
| 1997,MAJ Jeffrey A. Grasser | un | yes | un | yes | yes | un | yes | yes | un |
| 1997，Zain R B | yes | yes | yes | yes | yes | no | no | yes | yes |
| 2000，Mia HASHIBE | yes | un | yes | no | yes | yes | yes | no | yes |
| 2000，Rengaswamy Sankaranarayanan | yes | yes | yes | yes | yes | un | yes | yes | yes |
| 2000，T. Nagao | yes | no | yes | yes | no | no | yes | yes | not applicable |
| 2001，Dombi C | yes | no | yes | no | un | no | yes | yes | un |
| 2001，G. Campisi | yes | yes | un | no | yes | yes | yes | yes | no |
| 2001，H.C. Lin | yes | yes | no | yes | yes | no | yes | yes | yes |
| 2001，Nicola Pearson | un | un | un | no | un | no | yes | yes | yes |
| 2002，A Jainkittivong | no | un | no | no | no | no | un | yes | not applicable |
| 2002，Garcı´a-Pola Vallejo MJ | yes | yes | un | yes | yes | yes | yes | yes | no |
| 2003，Christian Scheifele | yes | yes | yes | yes | yes | no | yes | yes | un |
| 2003，I. Espinoza | yes | yes | no | no | yes | yes | yes | yes | un |
| 2003，Toru Nagao | un | no | no | yes | no | no | yes | yes | no |
| 2004，Paulo JoseÂ Benevides dos Santos | yes | yes | no | yes | yes | no | yes | yes | yes |
| 2005，Ching-Hung Chung | yes | yes | no | yes | yes | no | yes | yes | no |
| 2005，Eric Oakley | un | no | un | yes | no | no | yes | no | yes |
| 2006，TR Saraswathi | no | un | no | yes | un | un | un | yes | un |
| 2007，A. Ariyawardana | un | yes | yes | yes | yes | no | yes | yes | un |
| 2008，M Pentenero | un | un | no | yes | un | yes | yes | yes | un |
| 2008，Rushabh J Dagli | un | yes | no | yes | yes | no | yes | no | yes |
| 2008，Steven J. Thomas | yes | yes | no | yes | yes | no | yes | yes | yes |
| 2009，Ali-Rıza-İlker Cebeci | no | un | no | yes | un | no | yes | yes | not applicable |
| 2010，Hemantha K. Amarasinghe | yes | yes | no | yes | yes | yes | yes | no | yes |
| 2010,Ravi Mehrotra | yes | no | no | yes | yes | no | yes | no | un |
| 2010，Reto Morger | un | yes | no | no | yes | no | yes | yes | yes |
| 2010，Yi-Hsin Yang | yes | un | no | yes | un | no | yes | yes | yes |
| 2011，Amy Ming-Fang Yen | yes | un | yes | yes | no | un | yes | yes | yes |
| 2011，Raghavendra Byakodi | no | yes | yes | yes | un | un | yes | un | un |
| 2011，VC Carrard | yes | yes | no | yes | yes | yes | yes | yes | no |
| 2013，Agbor MA | un | no | un | yes | yes | no | yes | yes | yes |
| 2013，Fariborz Mansour Ghanaei | yes | yes | no | no | yes | un | yes | yes | un |
| 2013，Jairo Robledo-Sierra | no | yes | yes | yes | no | no | yes | yes | yes |
| 2013，Sushi Kadanakuppe | yes | yes | no | no | yes | yes | yes | yes | un |
| 2014，Mithra N. Hegde | no | un | no | yes | yes | yes | yes | yes | un |
| 2014，Queiroz | no | yes | yes | yes | yes | un | yes | no | un |
| 2014，Santhosh Vediyera Chandroth | un | yes | no | yes | yes | yes | yes | yes | yes |
| 2014，Y Hassona | no | un | no | yes | un | yes | yes | yes | not applicable |
| 2015，Chang-Cheng Chang | yes | yes | yes | yes | yes | yes | yes | yes | yes |
| 2015，Elizabeth L. Yanik | yes | yes | yes | yes | no | un | not applicable | yes | not applicable |
| 2015，Jinqiu Feng | yes | yes | yes | yes | yes | no | yes | yes | un |
| 2015，José Nicolau GHENO | yes | no | no | yes | no | un | yes | yes | not applicable |
| 2015，Nilesh Arjun Torwane | yes | yes | un | yes | yes | un | yes | no | yes |
| 2015，Sameer Rastogi | no | un | un | yes | yes | un | yes | yes | un |
| 2015，Viviani-Silva Araújo | un | yes | un | yes | yes | yes | yes | no | yes |
| 2017，Anzil KS Ali | un | yes |  | yes | yes | un | yes | yes | un |
| 2017，Divya Mehrotra | yes | yes | yes | yes | yes | un | un | yes | yes |
| 2017，He Liang | yes | no | yes | yes | no | no | no | yes | yes |
| 2017，Punith Shetty | un | yes | un | no | no | un | yes | yes | yes |
| 2018，Al-Maweri SA | no | yes | no | yes | yes | yes | yes | yes | yes |
| 2018，Andrej Aleksander Kansky | yes | yes | no | yes | yes | no | yes | yes | un |
| 2018，Christiana Madjova | no | yes | no | yes | yes | un | no | yes | not applicable |
| 2018，Nisha Rani Yadav | un | yes | no | yes | yes | un | yes | yes | yes |
| 2018,Shrikanth Muralidharan1 | un | yes | un | yes | yes | yes | un | yes | yes |
| 2018，Tepirou Chher | yes | yes | no | no | yes | un | yes | no | yes |
| 2019，Meghashyam Bhat | yes | yes | no | yes | yes | un | yes | yes | no |
| 2019，Sendhil Kumar | yes | no | no | yes | no | no | yes | no | not applicable |
| 2020，Carla Cruvinel Pontes | yes | no | no | yes | yes | yes | yes | yes | not applicable |
| 2020，Shuyun Ge | yes | yes | no | yes | yes | no | yes | yes | no |
| 2020，Ulla‑Maija Oivio | yes | yes | no | yes | un | no | yes | yes | no |
| 2021，Allan Vinícius Martins-de-Barros | yes | yes | un | no | yes | yes | yes | yes | yes |
| 2021，Stefan Kindler | yes | no | yes | yes | un | yes | yes | yes | no |
| 2021，Yunus G.Y. | un | no | un | yes | no | un | yes | yes | un |
| 2020，Afolabi Oyapero | un | yes | un | yes | yes | un | yes | yes | yes |
| 2022，Choudhury AR | un | yes | un | yes | yes | yes | yes | yes | yes |
| 2022，Denny E. Ceena | no | un | un | no | yes | un | yes | yes | not applicable |
| 2022，Sandeep Kumar | un | yes | un | yes | yes | yes | yes | no | un |
| 2022，Libin Benance Jacob | yes | yes | no | yes | yes | un | no | yes | yes |
